# The Role of Denosumab for Surgical Outcomes in Patients with Giant Cell Tumour of Bone: A Systematic Review

**DOI:** 10.3390/curroncol28020124

**Published:** 2021-03-22

**Authors:** Abha Gupta, Lisa Durocher-Allen, Snezana Popovic, Richard Tozer, Xiaomei Yao, Michelle Ghert

**Affiliations:** 1 Division of Haematology/Oncology, The Hospital for Sick Children, Toronto, ON M5G 1X8, Canada; abha.gupta@sickkids.ca; 2 Department of Oncology, McMaster University, Hamilton, ON L8S 4L8, Canada; durochl@mcmaster.ca (L.D.-A.); tozer@hhsc.ca (R.T.); yaoxia@mcmaster.ca (X.Y.); 3 Department of Pathology and Molecular Medicine, McMaster University, Hamilton, ON L8S 4L8, Canada; popovics@hhsc.ca; 4 Department of Health Research Methods, Evidence and Impact, McMaster University, Hamilton, ON L8S 4L8, Canada; 5 Department of Orthopedic Surgery, McMaster University, Hamilton, ON L8S 4L8, Canada

**Keywords:** denosumab, giant cell tumour of bone, surgical outcomes, systematic review

## Abstract

*Background:* The role of denosumab in patients with resectable giant cell tumour of bone remains unclear. We asked the following research question: for patients (aged ≥ 12 years) with resectable giant cell tumour of bone, what are the benefits and harms of denosumab compared with no denosumab in terms of (1) facilitation of surgery (operative time, blood loss), (2) disease recurrence, (3) pain control, (4) disease stability, and (5) adverse effects (e.g., malignant transformation, osteonecrosis of jaw, atypical femur fracture)? One previous systematic review addressed only one outcome—disease recurrence. Therefore, we undertook this new systematic review to address the above five outcomes. *Methods:* MEDLINE, EMBASE, PubMed, and Cochrane Database of Systematic Reviews databases were searched on June 30, 2020. *Results:* This systematic review included one previous systematic review and five comparative studies. Due to poor quality, non-randomized studies fraught with selection bias, it is difficult to determine if a significant difference exists in the outcomes for surgical giant cell tumour of bone with perioperative denosumab. There were no reported cases of adverse effects from denosumab. *Conclusion:* To date, there is insufficient evidence to understand the value of denosumab in the perioperative setting in patients with giant cell tumour of bone.

## 1. Introduction

Giant cell tumour of bone (GCTB) is an intermediate, osteoclastic, giant, cell-rich, primary bone tumour that is characterized by its locally aggressive and rarely metastasizing behaviour. GCTB typically develops at the meta-epiphysis of the long bones but can also present in pelvic and spinal sites, which prove more difficult to treat [1]. GCTB is locally aggressive due to the presence of numerous multinucleated osteoclast-type giant cells and mononuclear stromal cells expressing receptor activator of nuclear factor-KB (RANK) and RANK ligand (RANKL), which regulate osteoclast formation, migration, and survival [2]. This results in bone resorption, causing pain, limitations in range of motion, joint effusion, synovitis, and pathologic fracture in more extreme cases [3]. Surgical management of GCTB involves aggressive curettage of the lesion or removal of the affected bone en bloc.

Recent studies have suggested that patients treated with denosumab (DENO), a human monoclonal antibody RANKL inhibitor, experience favourable tumour responses and reduced need for surgery [4,5,6,7]. However, many of these studies are single-arm studies of patients on DENO (i.e., no comparison) or include patients who have ongoing treatment with DENO or who have completed DENO treatment but with a short follow-up [4,5,6]. There is some evidence that DENO treatment may cause the development of a new osseous tumour matrix and thickened cortical bone, possibly modifying a surgeon’s ability to curettage the lesion [8]. 

Although the initial phase 2 studies of DENO were compelling for the drug’s biologic effect on the tumour [9], the exact role of DENO in patients with resectable disease remains unclear. The purpose of this document is to provide evidence on the benefits and harms of DENO for the treatment of patients with GCTB, which will be used to inform the decisions of medical oncologists, orthopedic oncologists, pathologists, and other clinicians involved in the care of patients with GCTB, as well patients themselves. We asked the following research question: for patients (aged ≥ 12 years) with resectable GCTB, what are the benefits and harms of DENO compared with no DENO in terms of (1) facilitation of surgery (operative time, blood loss), (2) disease recurrence, (3) pain control, (4) disease stability, and (5) adverse effects (e.g., malignant transformation, osteonecrosis of jaw, atypical femur fracture)? One previous systematic review addressed only one outcome—disease recurrence. Therefore, we undertook this new systematic review to address the above five outcomes.

## 2. Methods

This systematic review has been registered on the PROSPERO website (International prospective registrar of systematic reviews, https://www.crd.york.ac.uk/prospero/ accessed on 10 March 2021) with the following registration number: CRD42020196392. The PRISMA reporting statements were followed.

### 2.1. Search for Existing Systematic Reviews

The following sources were searched for relevant systemic reviews: ECRI Guideline Trust Database, National Institute for Health and Care Excellence (NICE) evidence search, NICE (United Kingdom), Canadian Partnership Against Cancer Database, CMA Infobase, Scottish Intercollegiate Guideline Network, American Society of Clinical Oncology (ASCO), National Health and Medical Research Council, and Cancer Council Australia, Agency for Healthcare Research and Quality, Canadian Agency for Drugs and Technologies in Health, and PROSPERO databases. The following databases were also searched for relevant systemic reviews and original studies: MEDLINE, EMBASE, Cochrane Database of Systematic Reviews databases. The search took place on 30 June 2020, using varying terms of “denosumab”, “giant cell tumour of bone”, “benign fibrous histiocytoma of bone”, and “secondary aneurysmal bone cyst”. PubMed was also searched from January 2018 to 30 June 2020. The final search strategies are reported in Appendix A. The Clinicaltrials.gov website was searched for trials that were ongoing, unpublished, or incomplete from January 2015 to 19 August 2020. Conference proceedings from ASCO, European Society for Medical Oncology, and the Connective Tissue Oncology Society were searched from January 2017 to July 2020. 

### 2.2. Study Selection Criteria 

Systematic reviews were included if they addressed the research question with similar inclusion/exclusion criteria and the review had a low risk of bias for all four domains, as assessed with the Risk of Bias in Systematic Reviews (ROBIS) tool [10]. 

An article or abstract was included if it was a randomized control trial (RCT) (≥20 patients). If only RCTs with no or high risk of bias were available, then comparative studies (≥20 patients) were included if they used methods to control potential confounders such as multivariable analysis, propensity-score matching, or comparing patient characteristics to show no statistically significant differences between the comparison groups at baseline. An article was excluded if it was a single-arm study, letter, commentary, editorial, non-English full publications, tissue sample study, or abstract of a non-RCT.

A review of the titles and abstracts was done by one reviewer. For studies that warranted full-text review, one reviewer reviewed each article and discussed with the other Working Group members to confirm the final study selections. 

### 2.3. Data Extraction and Assessment of Risk of Bias

All included primary studies underwent data extraction by one reviewer, with all extracted data and information reviewed subsequently by an independent auditor. The risk of bias of included RCTs was assessed by the Cochrane Collaboration tools for randomized studies [11]. The risk of bias of included comparative non-randomized studies was evaluated with the Risk of Bias in non-randomized studies of interventions (ROBINS-I) [12].

### 2.4. Synthesizing the Evidence

Statistical analyses were executed with the statistical software package STATA version 15 [13]. When clinically and methodologically homogeneous results from two or more studies were available, a meta-analysis was conducted. When meta-analysis was inappropriate due to clinical heterogeneity, the results of each study were presented individually in a descriptive fashion. Ratios, including hazard ratios, were expressed with a ratio of <1.0 indicating a benefit for DENO treatment compared with the control. A two-sided significance level of α = 0.05 was assumed. The certainty of the evidence was assessed for the research question, considering risk of bias, inconsistency, indirectness, imprecision, and publication bias.

## 3. Results

**Figure 1:**
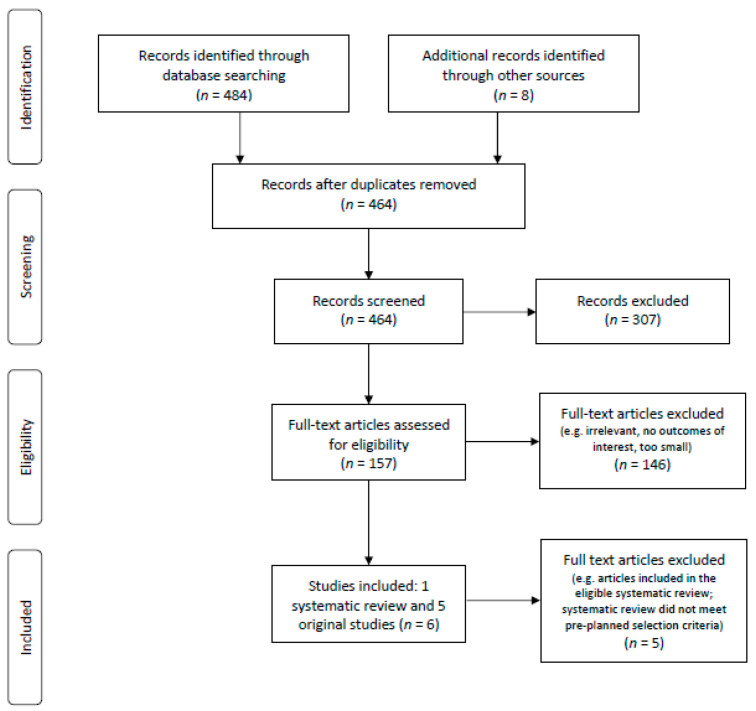
PRISMA flow diagram.

The PRISMA flow diagram of studies considered in the systematic review is shown in Figure 1.

### 3.1. Search for Systematic Reviews

Eighteen citations were identified from the systematic review search. From these, 15 were not relevant systematic reviews; one systematic review was excluded as it was a guideline’s systematic review and, at the time of this search, only the recommendations/charts had been updated and the literature search and discussion section indicated “update in progress” [14]; two systematic reviews [15,16] were assessed for risk of bias using the ROBIS tool (see Table 1) and only one met the pre-planned inclusion criteria as it had low risk of bias [16]. However, this eligible systematic review only reported one (i.e., disease recurrence) of five outcomes that we were interested in. Thus, we undertook this new systematic review to address all five outcomes.

### 3.2. Search for Primary Studies

The initial primary literature search, after removal of duplicates, resulted in 446 citations, from which 137 were identified to be eligible for full-text review. Among these, seven met our pre-planned study criteria [17,18,19,20,21,22,23] and their reference lists were manually search but no further eligible papers were found. A screen of conference abstracts yielded one abstract that met the study selection criteria [24]. Of these eight publications passing the initial screen, five underwent data extraction and were analyzed in this systematic review [17,18,19,20,21]. Three publications [22,23,24] did not undergo data extraction as they were detailed in the included systematic review [16]. 

Of the five articles, one was a retrospective, case-matched control study [17] and four were retrospective cohort studies [18,19,20,21]. All studies had very small number of patients who received DENO (seven to 30 patients).

Risk of bias assessments of five extracted studies are reported in Table 2 and the overall result for each study was a moderate to serious risk of bias [17,18,19,20,21]. The quality of aggregate evidence for every outcome was considered low to very low when considering risk of bias, inconsistency, indirectness, imprecision, and other factors altogether. Due to many of the studies being small, retrospective cohort studies where DENO administration was compared to a control group, there was an increase in bias as many reported different sample sizes between groups (with no power analysis), shorter time frame for those in the DENO group as it is a fairly new drug, and some patients treated in outside clinical centers. Due to clinical heterogeneity, meta-analyses were inappropriate for any outcomes. Table 3 summarizes the characteristics of these five included studies.

### 3.3. Outcomes

All articles that met inclusion criteria and had data extracted focused on patients with GCTB.

#### 3.3.1. Facilitation of Surgery/Reduced Surgical Morbidity

Results for facilitation of surgery/reduced surgical morbidity can be found in Table 3. Lim et al. compared patients receiving no DENO, adjuvant DENO, and both adjuvant and neoadjuvant DENO, and found that mean operating time in minutes was less for patients receiving both neoadjuvant and adjuvant DENO (mean ± standard deviation (SD) = 181.2 ± 38.6 min) when compared with no DENO (199.4 ± 49.5 min) or adjuvant DENO (200.6 ± 69.8 min), but the difference did not reach statistical significance [18]. This study also found that preoperative DENO was associated with reduced blood loss during surgery (*p* = 0.008) [18]. Agarwal et al. noted in their study that DENO administration in GCTB patients facilitated surgical resection by hardening the tumour and the bony shell, potentially reducing the risk of inadvertent contamination during separation of the neurovascular bundle or tendons from the tumour margin, although it increased the rate of recurrence [17]. While Medellin et al. observed that the use of DENO consolidated the peripheral rim and facilitated excision in patients presenting with fractures from GCTB, they also found that DENO neoadjuvant administration was associated with prolonged wait times before proceeding with surgery compared with no DENO (61 weeks vs. 4 weeks, *p* < 0.001) [19]. It is important to note that the number of patients in the DENO group was very small (*n* = 7).

#### 3.3.2. Disease Recurrence

Tsukamoto et al. performed a systematic review of seven comparative studies to determine if preoperative DENO had an effect on local recurrence risk in GCTB patients treated with curettage versus those treated with curettage alone and if preoperative DENO duration was associated with local recurrence after curettage [16]. Of the patients who received preoperative DENO and curettage, the local recurrence rates ranged from 20% to 100% (overall *n* = 619 patients), while in the curettage-alone group, this ranged from 0% to 50% (overall *n* = 127 patients). This suggests that there is an increased local recurrence risk in the DENO group, but due to poor quality, non-randomized trials, a meta-analysis was not performed to determine if there was a difference. In terms of the association between the duration of preoperative DENO and the association of local recurrence, in three trials where preoperative DENO was no more than six months, the odds ratios of local recurrence between the DENO group and no DENO group were 1.07, 2.76, and 37.80. Where preoperative DENO duration was more than six months in four trials, the odds ratios for local recurrence between the DENO group and no DENO group were 0.60, 5.71, 7.75, 28.33.

Two current studies not covered in the included previous systematic review are presented in Table 3. In a retrospective cohort study, Tsukamoto et al. found that local recurrences were higher in GCTB patients with surgery plus neoadjuvant DENO than those with surgery alone (50% vs. 15%, *p* < 0.0001) [21]. Lim et al. compared patients in three different groups and reported a local recurrence rate of 33% (12/36) in the no DENO group, 22% (2/9) in the adjuvant DENO group, and 17% (3/17) in the neoadjuvant and adjuvant DENO group (*p* = not significant (NS)) [18].

#### 3.3.3. Pain Control

There were no studies that evaluated pain control in patients receiving DENO versus no DENO.

#### 3.3.4. Disease Stability/Control

A total of two studies reported results of disease stability/control for patients receiving DENO vs. no DENO (see Table 3). Lim et al. reported disease control rates of patients receiving no DENO (66.7%) versus adjuvant DENO (77.8%) versus neoadjuvant plus adjuvant DENO (87.5%; *p* = NS) [18]. Tsukamoto et al. reported that patients in the DENO and surgery group had partial response rates in 22 patients (73.3%) and stable disease in eight patients (26.7%), but no results were reported in the no DENO group [21].

#### 3.3.5. Adverse Effects

##### Malignant Transformation

Although not related to DENO treatment, Tsukamoto et al. examined GCTB patients receiving DENO administration and surgery versus patients receiving surgery alone and found that patients in the two groups had similar lung metastasis rates with benign histology (3.3% versus 4.7%; *p* = 0.589) [21]. Lim et al. reported that 2 of 17 (3.2%) patients who received both neoadjuvant and adjuvant DENO had malignant transformation, but patients without DENO or only with neoadjuvant therapy of DENO did not have malignant transformation [18].

##### Osteonecrosis of Jaw

There were no reported cases of osteonecrosis of the jaw in any of the identified studies.

##### Atypical Femur Fracture

There were no studies that reported atypical femur fractures in patients receiving DENO versus no DENO.

### 3.4. Ongoing, Unpublished, or Incomplete Studies

There were no ongoing, unpublished, and incomplete studies found in The National Cancer Institute Clinical Trials Database (http://www.clinicaltrials.gov/ accessed on 19 August 2020) that met the inclusion criteria of this study. The search was conducted on 19 August 2020.

## 4. Discussion

DENO has garnered significant interest in the orthopaedic oncology field as a possible surgical adjuvant for giant-cell-rich lesions such as GCTB. The current systematic review of five original papers and one prior systematic review describes differences in perioperative outcome for patients with GCTB receiving DENO or not. Neoadjuvant DENO administration was associated with a shorter (not statistically significant) mean operating time than in patients receiving no DENO [18]. Further, neoadjuvant DENO resulted in less blood loss during surgery [18], more tumour and bony shell hardening [17], more new bone formation around and partially inside the lesion [20], and consolidated the peripheral rim and facilitated excision [19]. The previous systematic review found that patients receiving preoperative DENO prior to curettage had an increased risk of local recurrence compared with patients who received curettage alone [16]. Among two current studies not included in the previous systematic review, one supported this conclusion [21]; another did not reach statistically significant difference [18]. However, the poor quality of these studies, presence of selection bias, and lack of randomization make it very difficult to determine if a difference does indeed exist.

None of the included studies reported any cases of osteonecrosis of the jaw for either group. In regard to the development of benign histology metastasis, one study found that patients receiving DENO and surgery had similar incidence of lung metastases to patients receiving surgery alone, although this is not known to be related to DENO treatment [21].

This current systematic review only included RCTs or comparative studies, which included a control group where patients did not receive DENO. Thus, the initial large phase 2 trials of DENO were not included in the current analysis. This systematic review was limited since the quality of aggregate evidence for every outcome was low to very low. Moreover, there were no studies that evaluated pain control in patients receiving DENO versus no DENO, and short follow-up time (median 27 to 85 months) may be insufficient to accurately report malignant transformation of GCTB or osteonecrosis of jaw. Finally, when disease is deemed unresectable, DENO may be the only available option for clinicians and this systematic review did not address this issue in detail.

## 5. Conclusions

To date, there is insufficient comparative evidence to understand the value of DENO in the perioperative setting in patients with GCTB. Well-designed, prospective, comparative studies or RCTs are expected to better answer this research question.

## Figures and Tables

**Table 1:** Risk of Bias in Systematic Reviews (ROBIS) evaluation of included systematic reviews.

Study	Domain 1: Study Eligibility Criteria	Domain 2: Identification and Selection of studies	Domain 3: Data Collection and Study Appraisal	Domain 4: Synthesis and Findings	Overall Risk of Bias
Charest-Morin 2016 [15]	Unclear	Low	Unclear	Unclear	Unclear
Tsukamoto 2019a [16]	Low	Low	Low	Low	Low

**Table 2:** Risk of Bias in non-randomized studies of interventions (ROBIN-I) evaluation of included non-randomized studies.

Study	Bias Due to Confounding	Bias Due to Selection of Participants	Bias in Measurement of Interventions	Bias Due to Deviations of Interventions	Bias Due to Missing Data	Bias in Measurement of Outcomes	Bias in Selection of Reported Results	Overall Risk of Bias Judgement
Agarwal 2018 [17]	Serious	Serious	Moderate	Moderate	Moderate	Moderate	Moderate	Serious
Lim 2020 [18]	Serious	Moderate	Moderate	Serious	Moderate	Moderate	Moderate	Serious
Medellin 2018 [19]	Moderate	Serious	Moderate	Serious	Moderate	Serious	Moderate	Serious
Scoccianti 2018 [20]	Moderate	Moderate	Moderate	Moderate	Moderate	Moderate	Moderate	Moderate
Tsukamoto 2019b [21]	Moderate	Serious	Moderate	Serious	Moderate	Serious	Moderate	Serious

**Table 3:** Studies comparing DENO administration vs. no DENO administration.

Author and Study Type	Patient Population; Mean/ Median Age; Median Follow-Up Time	Arms or Comparisons	Number of Pts Analyzed	Facilitation of Surgery/Reduced Morbidity after Surgery	Disease Recurrence	Pain Control	Disease Stability/ Control	Adverse Effects
Agarwal 2018, Case-matched control [17]	54 pts with primary or recurrent GCTB located in the axial skeleton, appendicular skeleton, or distal tibia and sacrum; 32 (17–67) yr; 27–60 mths	Group 1: Neoadjuvant DENO 120 mg every month for 4 mths with additional doses of 120 mg on d8, d15 during 1st mth only	25	DENO aided surgical resection by hardening the tumour and the bony shell	Group 1: 11 (44%) in curettage, Group 2: 7 (21%), OR = 3.03 (95% CI 0.96 to 9.54), *p* = 0.085	NR	NR	No osteonecrosis of jaw
		Group 2: Surgery alone	34	NR				
Lim 2020, Retro cohort [18]	64 pts with sacral GCTB; 34 (11–65) yr; 48 (12–91) mths	Group 1: Surgery alone	36	Mean operating time (mins (SD)): 199.4 (49.5) vs. 200.6 (69.8) vs. 181.2 (38.6), *p* = NS. Blood loss during surgery (ml (SD)): 1715 vs. 1600 vs. 1418, *p* = 0.008	Local recurrence (n): 12 vs. 2 vs. 3. 1 yr RFS (%): 86.1 vs. 100 vs. 94.1, *p* = NS. 2 yr RFS (%) 72.2 vs. 100 vs. 86.3, *p* = NS. 3 yr RFS (%) 69.4 vs. 75.0 vs. 69.0, *p* = NS	NR	Local control rate: 66.7% vs. 77.8% vs. 87.5%, *p* = NS	NR
		Group 2: Adjuvant DENO 120 mg mthly. Continuation based on progress.	9					No osteonecrosis of jaw
		Group 3: Neoadjuvant DENO 120 mg d1, d8 and d15 with additional doses on d28 and every 4 wks, if required; Adjuvant: DENO 120 mg mthly, continuously based on progression.	17					No osteonecrosis of jaw, Malignant transformation: 3.2%
Medellin 2018, Retro Cohort [19]	120 patients with GCTB located in the femur and other bones; 33 (14–86) yr; 75 (12–301) mths	Group 1: Neoadjuvant DENO: 120 mg wk 1,2,3,5 and mthly until surgery. Mean duration of denosumab treatment prior to surgery was 8.9 (3–19) mths.	7	Mean time interval until initial surgery (Group 1 vs. Group 2): 61 wks (13–134) vs. 4 wks (0–19), *p* < 0.001. After initial surgery n = 41 (41%) in Group 2 required further surgical intervention. No data in Group 1.	Multivariate analysis showed DENO associated with higher risk of local recurrence (HR 3.2, 95% CI 1.07–9.55, *p* = 0.037)	NR	NR	No significant adverse effects that warranted cessation of DENO
		Group 2: Surgery alone	100					NR
Scoccianti 2018, Retro Cohort [20]	97 pts with GCTB located at the distal femur, distal tibia, distal radius and sacrum, proximal humerus, distal humerus, finger phalanx, iliac wing, proximal tibia, patella; 42 (17–66) yr; 27–39 mths	Group 1: Neoadjuvant DENO: 120 mg weekly for 3 wks, then monthly for 3 mths, then surgery	12	All showed new bone formation around and partially inside the lesion.	5 (42%) pts, Median 23 (7–54) mths post-surgery	NR	NR	No malignant transformation or osteonecrosis of jaw
		Group 2: Surgery alone	9	Curettage was considered feasible already at presentation.	1 (11%) pt, 14 mths post-surgery			NR
Tsukamoto 2019b, Retro Cohort [21]	411 pts with GCTB located in the distal radius and other sites such as the fibula, distal ulna, proximal radius, scapula, and patella; 29 (23–41) yr; 85 (IQR 54–124)	Group 1: Neoadjuvant DENO 120 mg once weekly for first mth and then once a mth for 6–9 mths, then surgery	30	NR	15 (50%) pts vs. 58 (15.2%) pts, *p* <0.0001	NR	Partial response: 22 (73.3%) pts, stable disease: 8 (26.7%) pts	1 (3.3%) pt experienced lung metastases vs. 18 (4.7%) pts, *p* = 0.589
		Group 2: Surgery alone	381				Not applicable	

CI = confidence interval; d = day; DENO = denosumab; GCTB = giant cell tumour of bone; HR = hazard ratio; IQR = interquartile range; mins = minutes; ml = millilitre; mthly = monthly; mths = months; NR = not reported; NS = not significant; OR = odds ratio; pts = patients; Retro = retrospective; Retro = retrospective; RFS = recurrence-free survival; SD = standard deviation; vs. = versus; wks = weeks; yr = years.

## Data Availability

Data sharing not applicable.

## Appendix A. Literature Search Strategy

### Appendix A.1. Giant Cell Tumour of Bone

Embase:

1. exp osteoclastoma/
2. osteoclastoma.mp.
3. (giant cell tumo$r adj4 bone).mp.
4. exp denosumab/
5. (denosumab or amgiva or prolia or xgeva or amg_162 or amg162).mp.
6. (1 or 2 or 3) and (4 or 5)

Medline:

1. exp “giant cell tumor of bone”/
2. (giant cell tumo$r adj4 bone).mp.
3. exp denosumab/
4. (denosumab or amgiva or prolia or xgeva or amg_162 or amg162).mp.
5. (1 or 2) and (3 or 4)
6. remove duplicates from 5

### Appendix A.2. Benign Fibrous Histiocytoma of Bone and Secondary Aneurysmal Bone Cyst

Embase:

1. exp osteoclastoma/
2. osteoclastoma.mp.
3. (giant cell tumo$r adj4 bone).mp.
4. (benign fibrous histiocytoma).mp.
5. exp bone cysts, aneurysmal/
6. (aneurysmal bone cystS).mp.
7. exp denosumab/
8. (denosumab or amgiva or prolia or xgeva or amg_162 or amg162).mp.
9. (4 or 5 or 6) not (1 or 2 or 3)
10. 9 and (7 or 8)
11. Remove duplicates from 10

Medline:

1. exp “giant cell tumor of bone”/
2. (giant cell tumo$r adj4 bone).mp.
3. exp Histiocytoma, Benign Fibrous/
4. Exp Bone Cysts, Aneurysmal/
5. (aneurysmal bone cyst$).mp.
6. exp denosumab/
7. (denosumab or amgiva or prolia or xgeva or amg_162 or amg162).mp.
8. (3 or 4 or 5) not (1 or 2)
9. 8 and (6 or 7)
10. Remove duplicates from 9
